# Thermochemical Liquefaction as a Cleaner and Efficient Route for Valuing Pinewood Residues from Forest Fires

**DOI:** 10.3390/molecules26237156

**Published:** 2021-11-26

**Authors:** Diogo Goncalves, Sofia Orišková, Sandro Matos, Henrique Machado, Salomé Vieira, David Bastos, Daniela Gaspar, Ricardo Paiva, João Carlos Bordado, Abel Rodrigues, Rui Galhano dos Santos

**Affiliations:** 1 CERENA-Centre for Natural Resources and the Environment, Instituto Superior Técnico, Av. Rovisco Pais, 1049-001 Lisboa, Portugal; diogo.azevedo.goncalves@ist.utl.pt (D.G.); sofia.oriskova@tecnico.ulisboa.pt (S.O.); sandro.matos@tecnico.ulisboa.pt (S.M.); henrimac97@gmail.com (H.M.); salomevieira@tecnico.ulisboa.pt (S.V.); ghostedd@hotmail.com (D.B.); danielafbgaspar@tecnico.ulisboa.pt (D.G.); jcbordado@ist.utl.pt (J.C.B.); 2 WOODCHEM SA, Estrada das Moitas Altas, 2401-902 Leiria, Portugal; 3 INIAV, Ministry of Agriculture, 2780-159 Oeiras, Portugal; r.paiva1@sapo.pt (R.P.); abel.rodrigues@iniav.pt (A.R.); 4 IDMEC—Instituto de Engenharia Mecânica, Instituto Superior Técnico, Av. Rovisco Pais, 1049-001 Lisboa, Portugal

**Keywords:** pinewood, forest wildfire, liquefaction, bio-oil

## Abstract

Biomass thermochemical liquefaction is a chemical process with multifunctional bio-oil as its main product. Under this process, the complex structure of lignocellulosic components can be hydrolysed into smaller molecules at atmospheric pressure. This work demonstrates that the liquefaction of burned pinewood from forest fires delivers similar conversion rates into bio-oil as non-burned wood does. The bio-oils from four burned biomass fractions (heartwood, sapwood, branches, and bark) showed lower moisture content and higher HHV (ranging between 32.96 and 35.85 MJ/kg) than the initial biomasses. The increased HHV resulted from the loss of oxygen, whereas the carbon and hydrogen mass fractions increased. The highest conversion of bark and heartwood was achieved after 60 min of liquefaction. Sapwood, pinewood, and branches reached a slightly higher conversion, with yields about 8% greater, but with longer liquefaction time resulting in higher energy consumption. Additionally, the van Krevelen diagram indicated that the produced bio-oils were closer and chemically more compatible (in terms of hydrogen and oxygen content) to the hydrocarbon fuels than the initial biomass counterparts. In addition, bio-oil from burned pinewood was shown to be a viable alternative biofuel for heavy industrial applications. Overall, biomass from forest fires can be used for the liquefaction process without compromising its efficiency and performance. By doing so, it recovers part of the lost value caused by wildfires, mitigating their negative effects.

## 1. Introduction

The extended use of petroleum as a source of energy and raw materials led to its depletion, high pollution levels, and a severe ecological footprint. Therefore, finding sustainable alternatives is critical. Lignocellulosic biomass arises as a possible option, as it is affordable and broadly available, however, it should not be a driver for further deforestation [1,2].

Several processes convert biomass into bio-oil, such as indirect liquefaction, e.g., fast pyrolysis, or direct liquefaction, e.g., thermochemical liquefaction and hydrothermal liquefaction. The latter is referred to as the hydrous depolymerisation process in an enclosed reactor that converts biomass into biocrude oil and chemicals at moderate temperature (200–400 °C) and high pressure (10–25 MPa) [3]. On the other hand, biomass thermochemical liquefaction is a chemical process that delivers a multifunctional liquid without costly pre-treatments [4]. This process enables the hydrolysis of the complex structure of lignocellulosic components into smaller molecules. This is achieved at atmospheric pressure, in the presence of an acid catalyst at temperatures ranging between 140 and 250 °C [5,6]. The advantage of both direct liquefaction processes is that they do not require drying of the biomass (pre-treatment step), which is essential to the fast pyrolysis process [7]. The bio-oil yield of the hydrothermal liquefaction is considerably lower compared to thermochemical liquefaction and fast pyrolysis [8,9]. Direct liquefaction results in higher quality bio-oil (high heating value, low moisture content), while pyrolysis leads to lower quality products [8,10,11].

Thermochemical liquefactions have been applied to a large number of feedstocks such as forest, agricultural, food, and industrial residues, e.g., eucalyptus [5,12,13], spruce [14], pinewood [9,15], poplar [16], cork powder [17,18], spent coffee beans [19], paper pulp sludge [11], swine manure [11], potato peels [20,21], wheat straw [14,22,23], and others.

Bio-oils from biomass thermochemical liquefaction can be converted into useful and valuable chemicals or used as a biofuel [16]. For instance, bio-oils can be used as adhesive components [24] or foams [25,26,27,28]. On the other hand, bio-oil application as a liquid biofuel with a high heating value has also been studied [10,11,16,29,30].

Thermochemical liquefaction has been studied, comprising a wide variety of solvents and catalysts. A Brønsted–Lowry acid catalyst is usually preferred, such as sulphuric acid [6]. However, some Lewis acids [31], zeolites [32], alkaline catalysts [32], and carbonates [33] are described as well. The use of p-toluenesulfonic acid is reported as delivering the highest conversion, i.e., bio-oil yield [31], without favouring the secondary condensation and repolymerisation reactions. Regarding solvents, glycerol, ethylene glycol, diethylene glycol, 2-ethylhexanol, polyethylene glycol, and some organic carbonates are among those widely used [6]. The thermochemical liquefaction is usually conducted using low biomass to solvent ratios from 1:3 to 1:5.

The production of lignocellulosic biomass in maritime pine stands must be carried out accordingly with a sustainable protocol of forest management with soil preparation, with thinnings, prunings, and wood harvesting for maximisation of wood productivity, carbon sequestration, and biodiversity [34,35]. In Portugal, as in other countries in the Mediterranean Basin, summer wildfires are a major problem which in 2017 claimed in total for the whole country about 537 kha [36] of burned areas. As such, the county of Pedrogão Grande was subjected in that year to fires with maximum energy release as high as 137 GW [37]. Additionally, the Leiria National Forest (LNF), a Portuguese public maritime pine forest with historical and economic relevance, extending over a sandy plane area of around 11,000 ha [36], was subjected to an intense passive crown fire in 2017 that burned approximately 86% [38] of the total area. The fire in LNF extended mainly to crown foliage with a likelihood pattern wherein the flame ascends to the tree crowns and further propagates between them [39]. Overall, this firing pattern preserves the wood structure, with hemicelluloses being the first component to react to flames, followed by cellulose, whilst lignin is the more difficult component to degrade [40].

The harvesting of dead trees leads to a glut of wood that may lower its prices [41]. The fire-affected wood can later suffer from bark beetles and fungi, with possible decreases in wood mechanical strength and financial value as well [42]. Burnt wood should be harvested immediately to retain most of its potential value and stored in controlled environments, aiming to lower the degree of decay and degradation [41].

The transformation of the burnt wood into wood fuel may increase its value and fuel efficiency [43] whilst possibly reducing the required storage space [44]. In this study, we evaluate the potential use of burned pinewood as feedstock for the liquefaction process. The results are compared with those from the liquefaction of unburnt pinewood shavings. The biomass and bio-oils are characterised by infrared spectroscopy, ultimate analysis, thermogravimetric analysis, and estimation of high heating value. The bio-oil’s energy densification ratio is also discussed.

## 2. Materials and Methods

**Figure 1:**
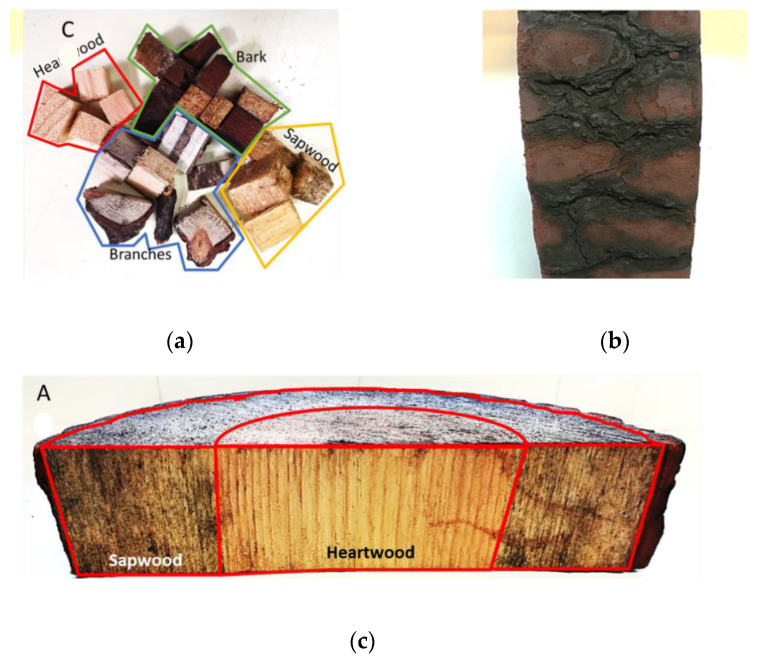
(**a**) Cubed samples and branches; (**b**) burnt bark; (**c**) slice of the burnt pinewood.

Several biomaterials were used as feedstock for the liquefaction process. Pinewood shavings (hereafter referred to as pinewood) were used as reference biomass. The Leiria National Park kindly supplied samples of slices of burned pinewood obtained from the fires of 2017. For liquefaction, five fractions were considered: bark, sapwood, heartwood, branches (Figure 1), and commercial pinewood shavings. The pine branches were collected from the forest ground.

Firstly, the biomass samples were cut into small cubes (Figure 1c) and dried in an oven at 110 ± 3 °C for 24 h. The drying process was conducted to facilitate the characterisation since it has no impact on the liquefaction process. Afterwards, the cubes were shredded in a Retsch© SM 2000 mill equipped with a 4 mm sieve to increase the contact surface area between the solvent and the feedstock. The reagents were: 2-ethylhexanol, purity ≥ 99%, p-toluenesulfonic acid (PTSA), purity ≥ 99%, from Sigma-Aldrich, and acetone, technical grade, acquired from a local gross supplier.

### 2.1. Liquefaction Procedure

The procedure consisted of a moderate acid-catalysed liquefaction process, in which the reaction occurs through a solvolysis reaction. The temperature was set based on previous studies [9]. Higher temperatures may increase unsoluble materials [15,41], humins, and solvent loss by evaporation [5]. The process occurred at 160 °C during the predetermined reaction time. A glass reactor was fed with the biomass samples and solvent, delivering a solution with a solvent:biomass ratio of 5:1. The solvent used was 2-ethylhexanol (2-EH), and the weight of biomass was based on its dry base. The mass of catalyst, PTSA, was 3% (*w*/*w*) of the mass of solvent and feedstock.

**Figure 2:**
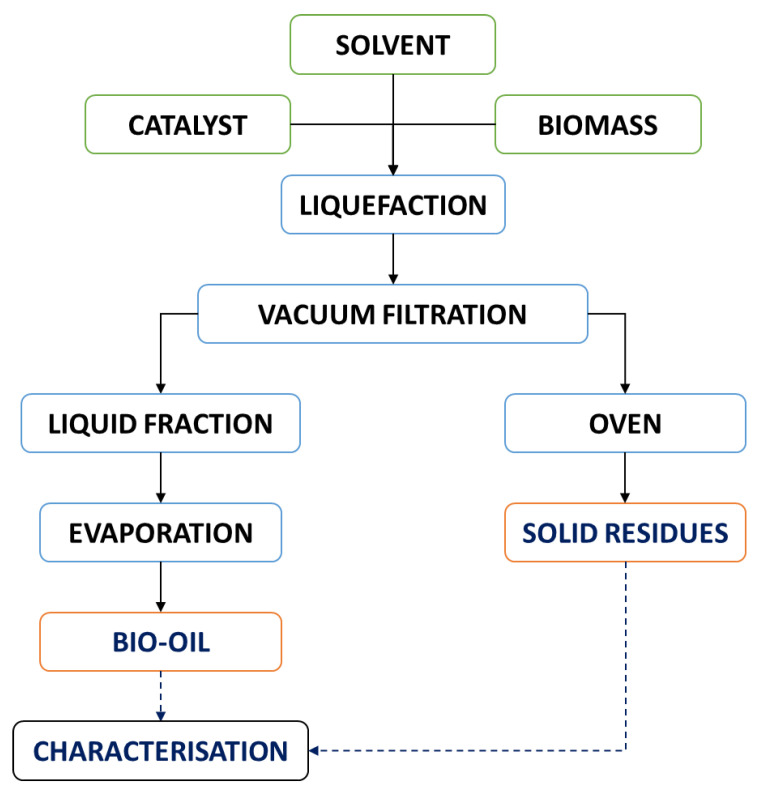
Liquefaction process flowchart of the pinewood samples.

Figure 2 depicts the overall procedure flowchart. After the set time, the reactor was cooled down to room temperature to be further subjected to vacuum-assisted filtration. The solid residues were washed with acetone to remove any bio-oil residue still present in the solids. The solid residues were dried in the oven at 110 ± 3 °C for 24 h.

Since the fraction of gaseous products appears in reduced amounts, it was neglected [15]. The solid residues encompass unreacted biomass and insoluble products that resulted from secondary condensation and repolymerisation reactions. Thus, the bio-oil yield, i.e., biomass conversion, was calculated based on the solid residue according to Equation (1).

Biomass yield (%) = (1 − *m*_s0_/*m*_si_) × 100 (1)

where *m*_si_ is the mass of dry biomass fed to the reactor, in grams, and *m*_s0_ is the mass of solid residues obtained at the end of the process, in grams.

The solvent of the bio-oil samples was removed using a BUCHI R-215 rotary evaporator, with a BUCHI B-491 heating bath, under a vacuum achieved by a BUCHI V-700 pump, with monitorisation with a BUCHI V-850 vacuum controller. The heating bath temperature was set at 100–120 °C, the rotation was at 80 rpm. The lowest recorded pressure during the process was 2.6 kPa. The solvent removal lasted for about 120 min.

### 2.2. Fourier Transformed Infrared (FTIR-ATR) Analysis of Biomass and Bio-Oil

The FTIR-ATR analysis was performed on a Perkin Elmer Spectrum Two spectrometer (Waltham, MA, USA) using a diamond ATR crystal. The spectra were captured from 4000 to 600 cm^−1^ and treated in Perkin Elmer Spectrum IR software.

### 2.3. Elemental Analysis and Higher Heating Value (HHV)

The chemical composition of the biomass and feedstock regarding carbon, hydrogen, and nitrogen (dry ash-free basis) was investigated by a LECO TruSpec CHN analyser, whilst a LECO CNS2000 analyser determined sulphur. Biomass and its derivatives contain mostly C, H, O elements, which summed up to approximately 97–99%. Additional elements, like sulphur and nitrogen, were present in negligible amounts, below the detection limit, and thus were difficult to measure or quantify [9,45]. The oxygen content was assessed according to Equation (2):

O (%) = 100 − C (%) − H (%)(2)

The higher heat values (HHVs) were estimated using correlations disclosed in the literature. Regarding the HHV of biomass, Equation (3) was used as disclosed by Yin et al. [46]. The HHV of bio-oils was assessed via Equation (4) by Mateus et al. [47]. The authors disclosed that the model was specifically developed for estimating the HHV of bio-oils obtained via thermochemical liquefaction. Both models presented a very low mean absolute error.

HHV (MJ/kg) = 0.2949C + 0.8250H(3)

HHV (MJ/kg) = 0.363302C + 1.087033H − 0.1009920(4)

The energy densification ratio (EDR), a dimensionless indicator, was calculated according to Equation (5):

EDR = HHV_bio-oil_/HHV_biomass_ (5)

where HHV_bio-oil_ and HHV_biomass_ are the higher heating values of bio-oil and biomass samples, respectively.

### 2.4. Thermogravimetric Analysis (TGA)

Thermogravimetric analysis was employed to study the samples’ behaviour at temperatures of interest. Bio-oils and solid residues of thermochemical liquefaction of each type of biomass were examined using Hitachi-STA7200 equipment. The analysis was performed at 25–600 °C in a N_2_ atmosphere, with a 100 mL/min flow and a heating rate of 5 °C/min.

## 3. Results and Discussion

### 3.1. Chemical Characterisation of Biomass Feedstock

The biomass was characterised before the liquefaction process. Table 1 presents the biomass’s chemical composition, moisture, and heating value. Even though biomass drying is usually non-essential in the liquefaction process, we did employ this pre-treatment step due to water usage during the firefight and the variability among the samples’ moisture contents. As shown in Table 1, the average moisture content after drying ranged between 2.97% for the bark and 25.18% for the branches. The elementary chemical composition was similar for all biomasses. The carbon, hydrogen, and oxygen contents ranged within the intervals 45.83–46.90%, 5.80–6.10%, and 47.30–48.90%, respectively (Table 1). The content of nitrogen and sulphur in the biomass was below the detection limit, confirming that these components can be neglected, as previously reported [9,45]. As the chemical composition of the different types of biomasses was similar, so were the HHV values, ranging between 18.25 and 18.77 MJ/kg.

### 3.2. Biomass Liquefaction

The purpose of this work was to evaluate the possibility of liquefying burnt pinewood biomass sourced from maritime pine trees after being caught in a wildfire in the Leiria National Forest in 2017. The flames influenced the bark (the trees’ outer layer) most significantly, hence the burned wood’s commercial value would tendentially decrease after the wildfire.

To evaluate the influence of the fire on the yield of liquefaction, we studied five biomass samples with reaction periods ranging from 0 to 300 min. Pinewood served as a reference, following previous work from Amado et al. [9].

**Figure 3:**
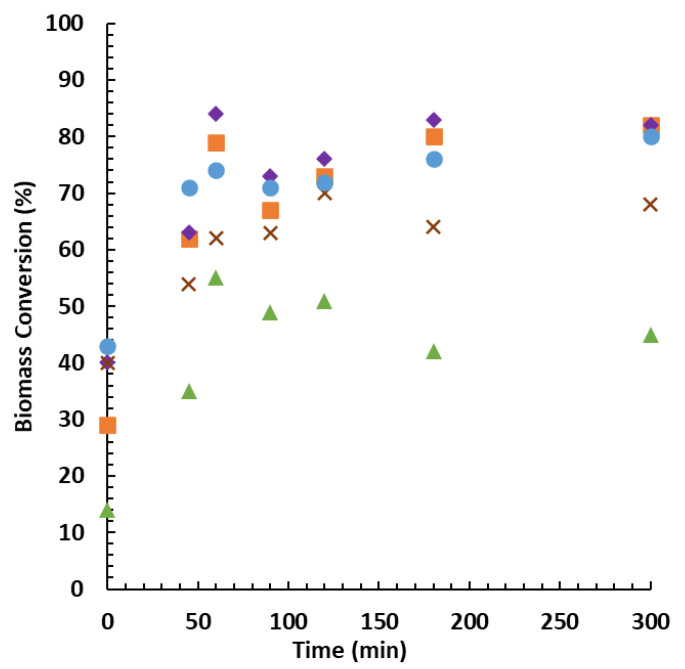
Biomass conversion rates by reaction time for each feedstock type: bark (▲), heartwood (**♦**), sapwood (**■**), pinewood (**●**), and branches (×).

The zero time (*t* = 0) was considered when the mixture reached the predetermined temperature of 160 °C. During the process, there was minimal gaseous fraction formed, thus we neglected its quantification. After the set time, the reactor was cooled down. No significant difference in the cooling times, for the different batches, was observed. The obtained results for the different biomasses are summarised in Figure 3. Biomass conversion of up to 84% was achieved. The conversion rates are greater than those obtained for fast pyrolysis (FP) [48,49,50,51] and hydrothermal liquefaction (HTL) [52,53].

Overall, the process resulted in the production of bio-oil from all tested types of feedstocks. Heartwood led to the highest conversion followed by sapwood, pinewood, branches, and bark (see Figure 3). The conversions at the zero time resulted from the liquefaction that occurred during the heating time.

The conversions of pinewood biomass reach a local maximum yield at 60 min. As previously reported [54,55,56,57], the branches present an exception, as their maximum yield is achieved after 120 min. After reaching the maximum conversion, a solid residue is formed, leading to the increase in the insoluble solid fraction. These solids (referred to as tar type and humin content) are commonly associated with recondensation reactions of decomposition products. This phenomenon leads to a decrease in the liquid fraction yields. In the case of prolonged reactions, the remaining biomass continues to liquefy, increasing the conversion. Regarding the bark, it must be pointed out that its lower conversion may be due mainly to the recalcitrance that is usually associated with bark, which hampers the hydrolysis of woody fibres [58,59]. The better behaviour in the process of the heartwood over sapwood may be explained by heartwood presenting better hydrothermal softening characteristics than sapwood [60].

Overall, the results are in accordance with other works using PTSA as a catalyst. This catalyst leads to good conversion, while sulphuric acid tends to increase solid residues with extended process time [31]. Regarding the decomposition mechanism that occurs, it is believed that in holocellulose, the oxygen atoms in the glycosidic bonds are protonated by the acidic catalyst, followed by the hydrolysis of the glycosidic bond and the consequent formation of carbonium ions [61], which react further, leading to smaller moieties. On the other hand, lignin follows a similar mechanism to that occurring during the cleavage of ether and ester bonds [62].

Even though, in some cases, the results demonstrate that the increased processing time provides a modest improvement in the final conversions, shorter reaction periods lead to the best (or at least very good) conversion rates and additionally save on energy consumption during the reaction. These assays have demonstrated that wildfire-affected pinewood can produce bio-oil with the same conversion rates as non-affected biomasses described in other works [13,15,19,20,23,31]. Considering the achieved conversions, we can conclude that liquefaction is a possible option to recover and prevent part of the loss of value during forest fires.

### 3.3. FTIR-ATR Analysis for the Biomass and Bio-Oils

**Figure 4:**
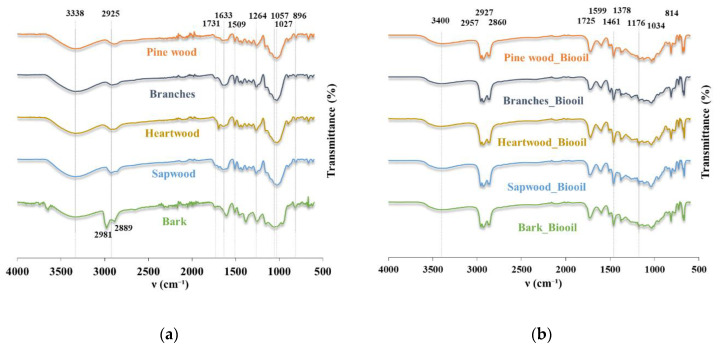
FTIR-ATR spectra of (**a**) biomasses obtained from Leiria National Park; (**b**) bio-oils from different feedstocks.

The FTIR spectra of the biomass samples and the bio-oils obtained from liquefaction are shown in Figure 4, with the most prominent peaks highlighted.

The functional groups of the lignocellulosic biomasses’ spectra are described in Table 2. Note that the presence of moisture in the biomass is verified in the spectra, with bands from 3600–3200 cm^−1^ attributed to O−H stretching bonds that may, to some extent, result from the water and/or hydroxyl groups. Moreover, the peak at 1633 cm^−1^, attributed to the OH bending of water, also reveals the presence of moisture. Those bands are especially noticeable in the spectra from branch biomass samples, which were the wettest, whilst drier samples (bark and heartwood) have much less evident bands. In contrast, in the spectra of the bio-oil (Figure 4b), the 1633 cm^−1^ peak is absent and the band in the 3600–3200 cm^−1^ region is less prominent, leading to the conclusion that water is not present in the bio-oil. Characteristic bands in biomass spectra (Figure 4a) were located in the 2950 cm^−1^ region, corresponding to the C−H stretching of CH_2_ and CH_3_. At 1731 cm^−1^, a weak peak corresponding to the C=O stretching is visible, which is associated with hemicellulose and lignin in raw biomass [6]. The C=C stretching peak is seen at 1599 cm^−1^, revealing the presence of aromatics moieties. At 1461 cm^−1^, the vibrational signal is mainly related to the C–H asymmetric deformation in –OCH_3_ and CH_2_ groups. Other peaks showing the evidence of aromatic derivatives are present at 1369 and 1264 cm^−1^, corresponding to aromatic C−H deformation and aromatic ring vibration, respectively. The former corresponds to the syringyl rings in lignin structure, while the latter is related to guaycyl rings. Conversely, peaks resulting from the carbohydrate components were identified at 896 cm^−1^, assigned to C−H out of plane vibration. A peak identified at 1027 cm^−1^ is related both to lignin and hemicellulose. The peaks resulting from biomass depolymerisation in the bio-oil spectra (Figure 4b) confirm the effectiveness of the liquefaction process. The carbonyl peak (1725 cm^−1^) of aldehydes or ketones relates to the conversion of cellulose/hemicellulose into levulinic acid and furfural [63]. Regarding the presence of aromatic derivatives, some signals are present at 1599, 1378, and 1034 cm^−1^. At the same time, carbohydrate-derived compounds are identified at 1461, 1176, 1034, and 814 cm^−1^. Lastly, the peaks in the region 2850–2960 cm^−1^ are possibly related to the presence of solvent residues or their reaction with the biomass. Overall, the bio-oil spectra reflected the expected chemical transformations of burned fractions of pinewood biomass during liquefaction, with the formation of chemical derivatives.

### 3.4. Elemental Analysis and HHV Calculation of the Bio-Oils

The elemental analysis, estimated HHV, ultimate analysis of the bio-oils, and other fuels used for comparison purposes can be found in Table 3. The heating value of bio-oils exceeds or is very similar to the HHVs of coals while being lower by about 10 MJ/kg than the hydrocarbons. These tendencies are reflected in the O/C ratios, which are higher for bio-oils than for hydrocarbons, anthracite, or bituminous coal. As expected, the liquefaction process decreased the O/C ratios of bio-oil products, averaging ~0.28, by comparison with the equivalent ratios in biomass feedstock, averaging ~1 (Table 1). The tendencies for the H/C ratios are not as obvious, as the values for bio-oil are similar to the hydrocarbons and higher than those from coals.

**Figure 5:**
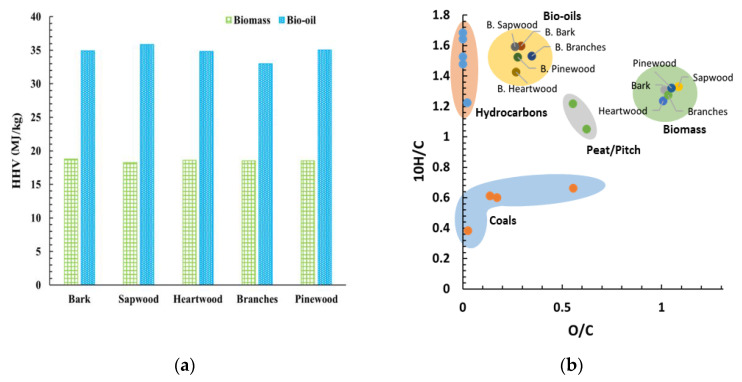
(**a**) HHV comparison of biomass and bio-oil; (**b**) van Krevelen diagram of the different samples.

Accordingly, Figure 5a shows a significant improvement of the HHV of the bio-oil compared to the biomass feedstock. The HHV of the bio-oils ranged from 32.96 to 35.85 MJ/kg, with the highest value recorded for the bio-oil from the sapwood. On average, liquefaction increases the HHV of the samples by 87%. Despite some variation among the calculated values of the HHV of the bio-oils from distinct biomass fractions, the differences were insignificant. Accordingly, the bio-oils’ energy gain is clear by evaluating the energy densification ratio, ranging from 1.86–1.96. This improvement is associated with the loss of oxygen (~61%) and water during liquefaction, increasing the carbon and hydrogen average mass fractions by ~50% and ~78%, respectively. Thermochemical liquefaction described in this work results in higher HHV values than the bio-oils obtained via fast pyrolysis. This phenomenon is explained by the higher oxygen content in the fast pyrolysis process [48,49,50,51,71,72,73].

The van Krevelen diagram shown in Figure 5b is a comprehensive way to compare samples with different oxygen and hydrogen contents. The chemical composition makes the bio-oils and the liquid hydrocarbon fuels the most alike, as observed in the diagram. The bio-oils’ data points shift from the biomasses’s, approaching hydrocarbons. This indicates a greater chemical similarity to the latter. Species with higher hydrogen content ignite more easily, hence burning “cleaner”.

The produced bio-oils present higher oxygen content than the hydrocarbon-based fuels, therefore they do not present an alternative to the applications where lower oxygen content is required. However, their quality is sufficient for industrial uses, serving as a biofuel replacement of coal.

The bio-oil moisture content is low (0.51–0.98%) since water evaporates during liquefaction. Additionally, the bio-oils from thermochemical liquefaction are much drier than those from fast pyrolysis [48,49,50,51,71,72,73,74].

### 3.5. Thermogravimetric Analysis of the Biomass and Bio-Oils

**Figure 6:**
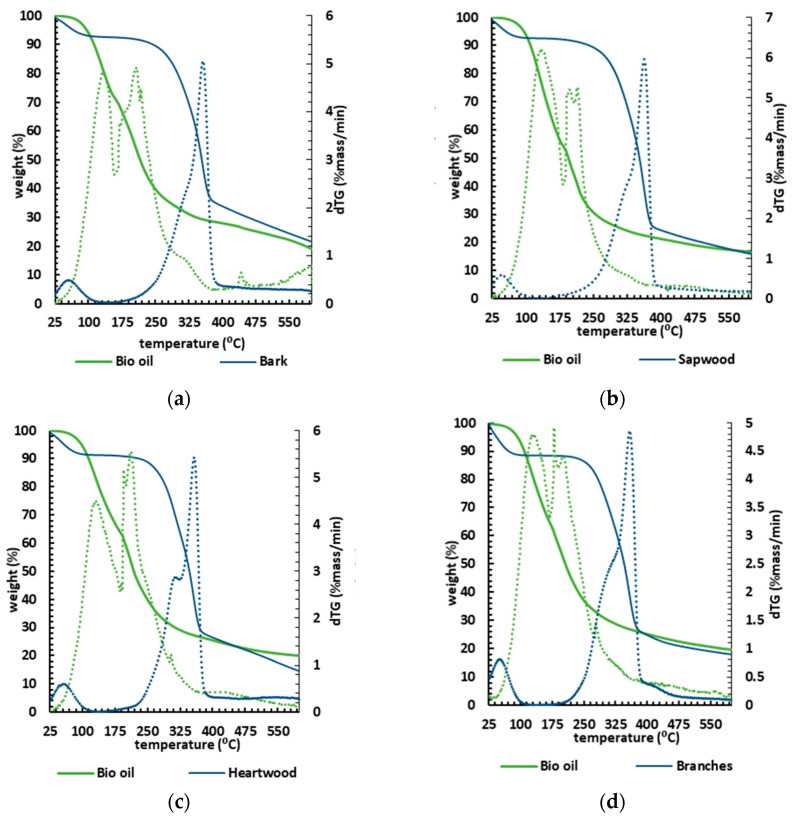

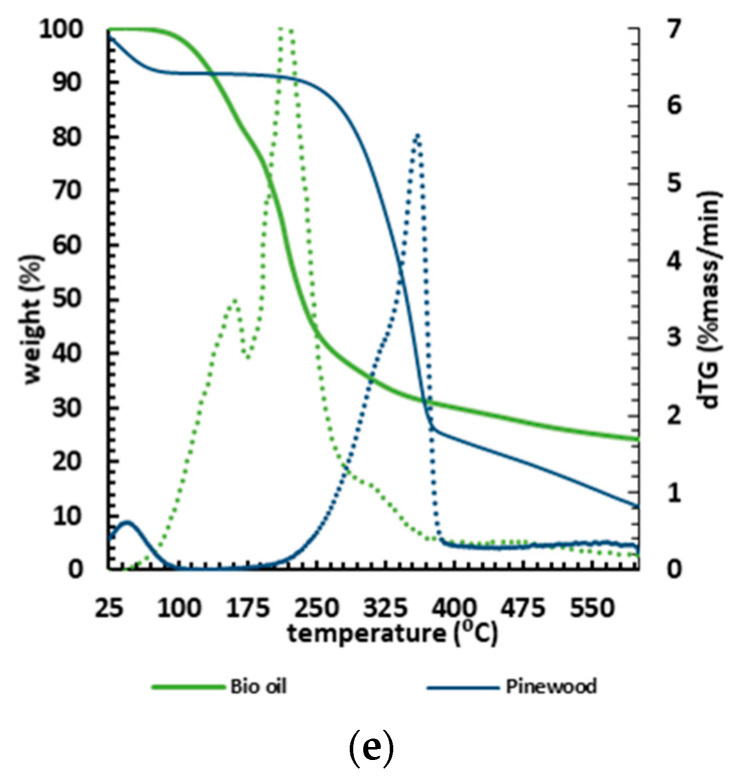
TGA (solid lines) and DTG (dashed lines) curves for the different biomasses: (**a**) bark; (**b**) sapwood; (**c**) heartwood; (**d**) branches; (**e**) pinewood, and their corresponding bio-oils.

Thermal stability is an important factor in selecting a material for a specific end use, predicting product performance and improving its quality. The TGA results (mass loss and DTG) are shown in Figure 6 in five graphs concerning each biomass and its respective liquefaction product. Each bio-oil sample consistently lost most of its mass at lower temperatures (100–300 °C) than their corresponding biomasses, which started to lose mass from 160 °C, systematically slowing down at 400 °C.

Considering the thermogravimetric analysis as a proxy to determine the biopolymeric composition in terms of cellulose, hemicellulose, and lignin [75], the TGA biomass analysis showed that the TGA curves were very similar, proving low variability in chemical composition within the tested samples.

A slight difference was spotted for heartwood, where the DTG curve showed a pronounced peak at 310 °C, corresponding to a slightly higher amount of hemicellulose and amorphous cellulose [76], though this minor variation is insignificant. Within all biomass samples, the initial loss stage begins at ~28 °C and ends at ~130 °C (Table 4).

The mass loss within the first range of the interval 25–135 °C averaged ~ 9% due to the removal of moisture and light volatile components from the biomass. The second stage (160–340 °C) was characterised by an average mass loss of ~ 30.4%. This stage, often described as active pyrolysis, is related to the degradation of hemicellulose and cellulose [76]. The third stage (340–400 °C) weight loss averaged about ~ 30.2% of mass loss (Table 4), with the maximum mass loss rate. The third stage is also considered an active pyrolysis stage. The thermal decomposition profiles of holocellulose and lignin are well defined [76], with the decomposition temperature ranges of hemicellulose, cellulose, and lignin being about 210–325, 310–400, and 160–900 °C, respectively. Even though the active pyrolysis stage degrades both hemicelluloses and cellulose, the simultaneous decomposition of lignin occurs in the second stage and predominantly in the third stage.

In the last stage (400–600 °C) there is a slight mass loss, attributed to the slow degradation of lignin to produce char as residue. This stage is also called passive pyrolysis and similar behaviour has been previously disclosed for other biomasses [76].

The TGA analysis shows that bio-oils from liquefaction are more volatile than the original biomasses, requiring lower temperatures to vaporise and decompose. Indeed, the lower energy required to combust them alongside the higher HHV (averaging about 35 MJ/kg) makes bio-oils from burned wood an attractive potential energy source for heavy industrial applications such as cement kilns.

The TGA curves also showed that the bio-oils decompose in a three-stage pattern (Table 4). The onset thermal temperature of thermal decomposition was ~50 °C, and the first stage, ending at 162–185 °C, corresponds to the lighter derivatives with an average loss of 37% of the samples’ mass. The first stage showed a slightly higher mass loss than the other two stages. The second stage, which persisted up to 300 °C, showed an average mass loss of ~33%, probably corresponding to the bio-oil’s heavier components. Note that bio-oil requires lower temperatures to decompose compared to the initial biomass. In the third stage (300–600 °C), one identifies a slight loss of mass, on average ~11%, attributed to the formation of non-degradable ash and carbon [77] resulting from the sample’s slow degradation. The DTG curves show that the bio-oil drops weight at an early stage, confirming the presence of a lighter product than the biomass counterparts. In fact, most of the mass loss is verified to occur below ~220 °C, hence strongly supporting their potential to be used in combustion processes [78].

## 4. Conclusions

Our work demonstrates that liquefaction of burned pinewood biomass from forest fires delivers similar conversion rates as non-burned wood. Sapwood and heartwood have the highest conversion rate, reaching 82% and 84%, respectively. The bark fraction, corresponding to the outer trunk layer, is the feedstock with the lowest conversion rate (55%), as it is the most affected by wildfire. As expected, the bio-oils from the burned biomass fractions showed lower moisture content and a higher HHV (ranging between 32.96 and 35.85 MJ/kg) than the initial biomasses. This increase in HHV resulted from oxygen loss and the increase in carbon and hydrogen mass fractions. The liquefaction after 60 min led to the highest conversion for the bark and heartwood. Regarding sapwood, branches, and pinewood shavings, slightly higher conversions (by up to 8%) were obtained after longer times, whilst consuming more energy.

Additionally, the van Krevelen diagram indicates that the produced bio-oils are closer and more chemically compatible (in terms of hydrogen and oxygen content) to the hydrocarbon fuels than to the initial biomass counterparts. Our results show that lower temperatures vaporise the lighter components of the bio-oils. Based on these results, we conclude that bio-oil from burned woods could be a viable alternative as an energy source for heavy industrial applications, such as cement kilns.

Overall, biomass from forest fires can be utilised in the liquefaction process without compromising its efficiency and performance. Through liquefaction, we can mitigate the negative effect of wildfires by recovering part of the lost value of burned woods.

## Figures and Tables

**Table 1:** Elemental analysis of pinewood biomass samples collected in Leiria National Forest.

	Sample	Moisture * (%)	Elemental Analysis (%)					HHV (MJ/kg)	O/C	10H/C
			S	C	H	N	O			
**Biomass**	Bark	2.97	<0.5	46.60	6.10	<2.0	47.30	18.77	1.015	1.309
	Sapwood	5.20	<0.5	45.10	6.00	<2.0	48.90	18.25	1.084	1.330
	Heartwood	14.70	<0.5	46.90	5.80	<2.0	47.30	18.62	1.009	1.237
	Branches	25.18	<0.5	46.30	5.90	<2.0	47.80	18.52	1.032	1.274
	Pinewood	12.00	<0.5	45.83	6.05	<2.0	48.12	18.51	1.050	1.320

* After drying.

**Table 2:** ATR-FTIR band assignment (characteristic bands) for the bio-oils and biomass spectra.

Wavenumber (cm^−1^)		Band Assignment	Compound/Group	Ref.
Biomass	Bio-Oil			
3338	3400	OH stretching	hydroxyl groups	[64]
2981 2925 2889	2957 2957 2860	CH2−, CH3− stretching	methylene and methyl groups from holocellulose and lignin	[9]
1731	1725	C=O stretching	ketones and esters	[54]
1633	--	OH bending	water	[65,66]
1599	1599	C=C stretching	aromatic ring	[64]
1461	1461	OCH3–, –CH2–, C−H	carbohydrates	[67]
1369	1378	aromatic C–H deformation	syringyl rings (from lignin)	[54]
1264	--		guaycyl rings	[68]
--	1176	aromatic ring vibration	cellulose	[69]
1027	1034	C−O−C asymmetric stretching	cellulose, hemicellulose, lignin	[68]
896	--	C−O, C=C, and C−C−O	hemicellulose	[68]
--	814	stretching	carbohydrates	[30]

**Table 3:** Moisture content, elemental analysis, and HHV of bio-oil produced from different types of biomasses after 60 min.

	Sample	Moisture * (%)	Elemental Analysis (%)					HHV (MJ/kg)	O/C	10H/C	EDR * (%)
			S	C	H	N	O				
**Bio-oils**	Bark	0.96	<0.5	68.79	10.99	<2.0	20.22	34.90	0.294	1.598	1.86
	Sapwood	0.98	<0.5	70.30	11.20	<2.0	18.50	35.85	0.263	1.593	1.96
	Heartwood	0.86	<0.5	70.90	10.10	<2.0	19.00	34.82	0.268	1.425	1.87
	Branches	0.84	<0.5	66.65	10.20	<2.0	23.15	32.96	0.347	1.530	1.78
	Pinewood	0.51	<0.5	69.95	10.66	<2.0	19.39	35.04	0.277	1.524	1.89
**Coals**	Anthracite [70]	--	0.83	91.60	3.50	1.60	2.39	35.63	0.026	0.382	--
	Lignite [70]	--	0.61	60.51	4.01	1.22	33.66	21.52	0.556	0.663	--
	Bituminous coal [70]	--	0.43	81.80	5.00	1.50	11.21	33.69	0.137	0.611	--
	Coal [70]	--	0.41	78.31	4.71	2.30	13.50	30.86	0.172	0.601	--
**Hydrocarbons**	Kerosene [70]	--	0.10	85.80	14.10	0.00	0.00	46.50	0.000	1.643	--
	Gasoline [70]	--	0.10	85.50	14.40	0.00	0.00	46.88	0.000	1.684	--
	Fuel oil 6 [70]	--	0.05	85.70	10.50	1.70	2.00	42.30	0.023	1.225	--
	Fuel oil 2 [70]	--	0.00	87.30	12.90	0.00	0.01	43.80	0.000	1.478	--
	Diesel [70]	--	0.30	86.50	13.20	0.00	0.00	45.70	0.000	1.526	--
	Pitch [70]	--	0.00	59.67	7.27	0.00	33.05	26.70	0.554	1.218	--
	Peat [70]	--	0.17	56.88	5.98	1.53	35.38	22.65	0.622	1.051	--

* EDR—energy densification ratio.

**Table 4:** TG temperatures and mass loss of the biomasses and bio-oils.

		1st Stage		2nd Stage		3rd Stage		4th Stage	
	**Sample**	**Temp. Range (°C)**	**Mass Loss (%)**	**Temp. Range (°C)**	**Mass Loss (%)**	**Temp. Range (°C)**	**Mass Loss (%)**	**Temp. Range (°C)**	**Mass Loss (%)**
**Biomass**	Bark	25–135	7	160–340	31	340–400	27	400–600	12
	Sapwood		7		33		35		8
	Heartwood		8		28		28		12
	Branches		12		28		29		7
	Pinewood		8		32		32		13
**Bio-oil**	Bark	50–162	30	162–300	37	300–600	14	---	---
	Sapwood	50–185	47	185–300	28		9		
	Heartwood	50–185	35	185–300	34		11		
	Branches	50–170	36	170–300	34		10		
	Pinewood	50–185	23	170–300	41		12		

## Sample Availability

No samples are available.

## References

[1] Hosonuma N., Herold M., De Sy V., De Fries R.S., Brockhaus M., Verchot L., Angelsen A., Romijn E. (2012). An assessment of deforestation and forest degradation drivers in developing countries. Environ. Res. Lett..

[2] Bailis R., Wang Y., Drigo R., Ghilardi A., Masera O. (2017). Getting the numbers right: Revisiting woodfuel sustainability in the developing world. Environ. Res. Lett..

[3] Zhang Y., Chen W.-T. (2018). Hydrothermal liquefaction of protein-containing feedstocks. Direct Thermochem. Liq. Energy Appl..

[4] Kržan A., Kunaver M. (2006). Microwave heating in wood liquefaction. J. Appl. Polym. Sci..

[5] Fernandes F., Matos S., Gaspar D., Silva L., Paulo I., Vieira S., Pinto P.C.R., Bordado J., dos Santos R.G. (2021). Boosting the Higher Heating Value of Eucalyptus globulus via Thermochemical Liquefaction. Sustainability.

[6] Condeço J.A., Hariharakrishnan S., Ofili O.M., Mateus M.M., Bordado J.M., Correia M.J.N. (2021). Energetic valorisation of agricultural residues by solvent-based liquefaction. Biomass Bioenergy.

[7] Abaide E.R., Ahmadzadeh H., Alzate C.A.C., Amiri H., Ang G.T., Avhad M.R., Biswas N., Burton E., Costa J.A.V., Da Silva P.P., Hosseini M. (2019). List of Contributors. Advanced Bioprocessing for Alternative Fuels, Biobased Chemicals and Bioproducts.

[8] Xiu S., Shahbazi A. (2012). Bio-oil production and upgrading research: A review. Renew. Sustain. Energy Rev..

[9] Amado M., Bastos D., Gaspar D., Matos S., Vieira S., Bordado J.M., dos Santos R.G. (2021). Thermochemical liquefaction of pinewood shaves—Evaluating the performance of cleaner and sustainable alternative solvents. J. Clean. Prod..

[10] Mateus M., Bordado J., dos Santos R.G. (2016). Potential biofuel from liquefied cork—Higher heating value comparison. Fuel.

[11] dos Santos R.G., Bordado J., Mateus M.M. (2016). Potential biofuels from liquefied industrial wastes—Preliminary evaluation of heats of combustion and van Krevelen correlations. J. Clean. Prod..

[12] Zhang H., Pang H., Shi J., Fu T., Liao B. (2012). Investigation of liquefied wood residues based on cellulose, hemicellulose, and lignin. J. Appl. Polym. Sci..

[13] Mateus M., Guerreiro D., Ferreira O., Bordado J., dos Santos R.G. (2017). Heuristic analysis of Eucalyptus globulus bark depolymerization via acid-liquefaction. Cellulose.

[14] Kunaver M., Jasiukaitytė E., Čuk N. (2012). Ultrasonically assisted liquefaction of lignocellulosic materials. Bioresour. Technol..

[15] Braz A., Mateus M.M., dos Santos R.G., Machado R., Bordado J.M., Correia M.J.N. (2019). Modelling of pine wood sawdust thermochemical liquefaction. Biomass Bioenergy.

[16] Mateus M., Vale M., Rodrigues A., Bordado J., dos Santos R.G. (2017). Is biomass liquefaction an option for the viability of poplar short rotation coppices? A preliminary experimental approach. Energy.

[17] Viana R.E.H., Dos Santos S.G., Oliveira A.C., Information P.E.K.F.C. (2015). Recovery of resistant bacteria from mattresses of patients under contact precautions. Am. J. Infect. Control..

[18] Soares B., Gama N., Freire C., Timmons A.B., Brandão I., Silva R., Neto C.P., Ferreira A. (2014). Ecopolyol Production from Industrial Cork Powder via Acid Liquefaction Using Polyhydric Alcohols. ACS Sustain. Chem. Eng..

[19] Soares B., Gama N., Freire C.S., Barros-Timmons A., Brandão I., Silva R., Neto C.P., Ferreira A. (2015). Spent coffee grounds as a renewable source for ecopolyols production. J. Chem. Technol. Biotechnol..

[20] Dos Santos R.G., Ventura P., Bordado J., Mateus M. (2017). Direct and efficient liquefaction of potato peel into bio-oil. Environ. Chem. Lett..

[21] Dos Santos R.G., Ventura P., Bordado J., Mateus M. (2016). Valorizing potato peel waste: An overview of the latest publications. Rev. Environ. Sci. Bio Technol..

[22] Wang H., Chen H.-Z. (2007). A novel method of utilizing the biomass resource: Rapid liquefaction of wheat straw and preparation of biodegradable polyurethane foam (PUF). J. Chin. Inst. Chem. Eng..

[23] Liang L., Mao Z., Li Y., Wan C., Wang T., Zhang L., Zhang L. (2006). Liquefaction of crop residues for polyol production. Bioresources.

[24] dos Santos R.G., Carvalho R., Silva E.R., Bordado J., Cardoso A.C., Costa M.D.R., Mateus M. (2016). Natural polymeric water-based adhesive from cork liquefaction. Ind. Crop. Prod..

[25] Dos Santos R.G., Acero N.F., Matos S., Carvalho R., Vale M., Marques A.C., Bordado J.C., Mateus M.M. (2017). One-Component Spray Polyurethane Foam from Liquefied Pinewood Polyols: Pursuing Eco-Friendly Materials. J. Polym. Environ..

[26] Lee S.-H., Teramoto Y., Shiraishi N. (2001). Biodegradable polyurethane foam from liquefied waste paper and its thermal stability, biodegradability, and genotoxicity. J. Appl. Polym. Sci..

[27] Gama N.V., Soares B., Freire C.S., Silva R., Brandão I., Neto C.P., Barros-Timmons A., Ferreira A. (2015). Rigid polyurethane foams derived from cork liquefied at atmospheric pressure. Polym. Int..

[28] Vale M., Mateus M., dos Santos R.G., de Castro C.N., de Schrijver A., Bordado J.C., Marques A.C. (2019). Replacement of petroleum-derived diols by sustainable biopolyols in one component polyurethane foams. J. Clean. Prod..

[29] Seljak T., Oprešnik S.R., Kunaver M., Katrašnik T. (2012). Wood, liquefied in polyhydroxy alcohols as a fuel for gas turbines. Appl. Energy.

[30] Mateus M., Gaspar D.F.B., Matos S., Rego A., Motta C., Castanheira I., Bordado J.M., dos Santos R.G. (2019). Converting a residue from an edible source (Ceratonia siliqua L.) into a bio-oil. J. Environ. Chem. Eng..

[31] Mateus M., Carvalho R., Bordado J., dos Santos R.G. (2015). Biomass acid-catalyzed liquefaction—Catalysts performance and polyhydric alcohol influence. Data Brief.

[32] Li Y., Luo X., Hu S. (2015). Lignocellulosic Biomass-Based Polyols for Polyurethane Applications.

[33] Karagöz S., Bhaskar T., Muto A., Sakata Y. (2004). Effect of Rb and Cs carbonates for Production of Phenols from liquefaction of wood biomass. Fuel.

[34] Oliveira A.C., Pereira J.S., Correia A.V. A Silvicultura Do Pinheiro Bravo. 2000. ISBN: 972-98308-2-7. https://www.centropinus.org/files/upload/edicoes_tecnicas/7ca9c5bad087cc24e889debc852ec75fdf1c2143.pdf.

[35] Brasov A., Nicolescu V.N. The Practice of Silviculture.

[36] Divisão de apoio à Gestão de Fogos Rurais (2020). 8° Relatório Provisório de Incêndios Rurais.

[37] (2017). Comissão Técnica Independente, Relatório Comunidade Independente—Análise e Apuramento dos Factos Relativos aos Incêndios que Ocorreram em Pedrogão Grande, Castanheiro de Pera, Ansião, Alvaiázere, Figueiró de Vinhos, Arganil, Góis, Penela, Pampilhosa da Serra, Oleiros e Sertã, entre. https://www.parlamento.pt/Documents/2017/Outubro/RelatórioCTI_VF.pdf.

[38] Beighley M., Hyde A.C. (2018). Gestão dos Incêndios Florestais em Portugal numa Nova Era Avaliação dos Riscos de Incêndio, Recursos e Reformas. https://www.isa.ulisboa.pt/files/events/pub/2018_Portugal-Wildfire-Management-in-a-New-Era_Portuguese.pdf.

[39] U.S. National Park Service (2015). Wildland Fire Spread and Suppression. https://www.nps.gov/articles/wildland-fire-spread-and-suppression.htm.

[40] Yang Z., Kumar A., Huhnke R. (2015). Review of recent developments to improve storage and transportation stability of bio-oil. Renew. Sustain. Energy Rev..

[41] UNECE/FAO (2020). Annual Market Review 2019–2020—Forest Products.

[42] Barkley Y.C. (2015). After the Burn: Assessing and Managing Your Forestland after a Wildfire, Moscow, Idaho. https://www.fs.usda.gov/rmrs/documents-and-media/after-burn-assessing-and-managing-your-forestland-after-wildfire.

[43] Johnson F.X., Pacini H., Smeets E. (2012). Transformations in EU Biofuels Markets under the Renewable Energy Directive and the Implications for Land Use, Trade and Forests.

[44] Food and Agriculture Organization of the United Nations Sustainable Forest Management (SFM) Toolbox; Wood Energy, (n.d.). http://www.fao.org/sustainable-forest-management/toolbox/modules/wood-energy/basic-knowledge/en/?type=111.

[45] Sheng C., Azevedo J. (2005). Estimating the higher heating value of biomass fuels from basic analysis data. Biomass Bioenergy.

[46] Yin C.-Y. (2011). Prediction of higher heating values of biomass from proximate and ultimate analyses. Fuel.

[47] Mateus M.M., Bordado J.M., dos Santos R.G. (2021). Estimation of higher heating value (HHV) of bio-oils from thermochemical liquefaction by linear correlation. Fuel.

[48] Mullen C., Boateng A.A. (2008). Chemical Composition of Bio-oils Produced by Fast Pyrolysis of Two Energy Crops. Energy Fuels.

[49] Ben H., Wu F., Wu Z., Han G., Jiang W., Ragauskas A.J. (2019). A Comprehensive Characterization of Pyrolysis Oil from Softwood Barks. Polymers.

[50] Oyebanji J., Okekunle P., Lasode O., Oyedepo S. (2018). Chemical composition of bio-oils produced by fast pyrolysis of two energy biomass. Biofuels.

[51] Chukwuneke J., Ewulonu C., Chukwujike I., Okolie P. (2019). Physico-chemical analysis of pyrolyzed bio-oil from swietenia macrophylla (mahogany) wood. Heliyon.

[52] Tian Y., Wang F., Djandja J.O., Zhang S.-L., Xu Y.-P., Duan P.-G. (2020). Hydrothermal liquefaction of crop straws: Effect of feedstock composition. Fuel.

[53] Jindal M.K., Jha M.K. (2016). Hydrothermal liquefaction of wood: A critical review. Rev. Chem. Eng..

[54] Mateus M.M., Ventura P., Rego A., Motta C., Castanheira I., Bordado J., Dos Santos R.G. (2016). Acid Liquefaction of Potato (Solanum tuberosum) and Sweet Potato (Ipomoea batatas) Cultivars Peels—Pre-Screening of Antioxidant Activity/Total Phenolic and Sugar Contents. Bioresources.

[55] Hassan E.B., Shukry N. (2008). Polyhydric alcohol liquefaction of some lignocellulosic agricultural residues. Ind. Crop. Prod..

[56] Pan H., Zheng Z., Hse C.Y. (2012). Microwave-assisted liquefaction of wood with polyhydric alcohols and its application in preparation of polyurethane (PU) foams. Eur. J. Wood Wood Prod..

[57] Rg D.S., Mm B.J.M., Jc B. (2015). Microwave-assisted Liquefaction of Cork—From an Industrial Waste to Sustainable Chemicals. Ind. Eng. Manag..

[58] Zhang C., Xu W., Yan P., Liu X., Zhang Z.C. (2015). Overcome the recalcitrance of eucalyptus bark to enzymatic hydrolysis by concerted ionic liquid pretreatment. Process. Biochem..

[59] Frankó B., Galbe M., Wallberg O. (2015). Influence of bark on fuel ethanol production from steam-pretreated spruce. Biotechnol. Biofuels.

[60] Li Z., Lu J., Cao J., Jiang J. (2020). Comparative Study of the Hydrothermal Softening Characteristics of Heartwood and Sapwood. For. Prod. J..

[61] Mun S.P., Jang J.P. (2009). Liquefaction of cellulose in the presence of phenol using p-toluene sulfonic acid as a catalyst. J. Ind. Eng. Chem..

[62] Zhai Q., Long F., Hse C.-Y., Wang F., Shupe T.F., Jiang J., Xu J. (2019). Facile Fractionation of Bamboo Wood Toward Biomass Valorization by p-TsOH-Based Methanolysis Pretreatment. ACS Sustain. Chem. Eng..

[63] Grilc M., Likozar B., Levec J. (2015). Kinetic model of homogeneous lignocellulosic biomass solvolysis in glycerol and imidazolium-based ionic liquids with subsequent heterogeneous hydrodeoxygenation over NiMo/Al_2_O_3_ catalyst. Catal. Today.

[64] Bui N.Q., Fongarland P., Rataboul F., Dartiguelongue C., Charon N., Vallée C., Essayem N. (2015). FTIR as a simple tool to quantify unconverted lignin from chars in biomass liquefaction process: Application to SC ethanol liquefaction of pine wood. Fuel Process. Technol..

[65] Zohdi V., Whelan D., Wood B.R., Pearson J., Bambery K., Black M.J. (2015). Importance of Tissue Preparation Methods in FTIR Micro-Spectroscopical Analysis of Biological Tissues: ‘Traps for New Users’. PLoS ONE.

[66] Zhuang J., Li M., Pu Y., Ragauskas A.J., Yoo C.G. (2020). Observation of Potential Contaminants in Processed Biomass Using Fourier Transform Infrared Spectroscopy. Appl. Sci..

[67] Popescu C.-M., Popescu M.-C., Singurel G., Vasile C., Argyropoulos D., Willför S. (2007). Spectral Characterization of Eucalyptus Wood. Appl. Spectrosc..

[68] Xu F., Yu J., Tesso T., Dowell F., Wang D. (2013). Qualitative and quantitative analysis of lignocellulosic biomass using infrared techniques: A mini-review. Appl. Energy.

[69] Yona A.M.C., Budija F., Kričej B., Kutnar A., Pavlič M., Pori P., Tavzes Č., Petrič M. (2014). Production of biomaterials from cork: Liquefaction in polyhydric alcohols at moderate temperatures. Ind. Crop. Prod..

[70] (2012). ECN, Phyllis2-Database for the Physico-Chemical Composition of (Treated) Lignocellulosic Biomass, Micro and Macroalgae, Various Feedstocks for Biogas Production and Biochar. Energy Res. Cent. Neth..

[71] Sadaka S., Boateng A.A. (2009). Pyrolysis and Bio-Oil. Agric. Nat. Resour..

[72] Balat M. (2010). Bio-Oil Production from Pyrolysis of Black Locust (Robinia pseudoacacia) Wood. Energy Explor. Exploit..

[73] Lehto J., Oasmaa A., Solantausta Y., Kytö M., Chiaramonti D. (2014). Review of fuel oil quality and combustion of fast pyrolysis bio-oils from lignocellulosic biomass. Appl. Energy.

[74] Lyu G., Wu S., Zhang H. (2015). Estimation and Comparison of Bio-Oil Components from Different Pyrolysis Conditions. Front. Energy Res..

[75] Rego F., Dias A.P.S., Casquilho M., Rosa F.C., Rodrigues A. (2019). Fast determination of lignocellulosic composition of poplar biomass by thermogravimetry. Biomass Bioenergy.

[76] Varma A.K., Mondal P. (2016). Physicochemical characterization and kinetic study of pine needle for pyrolysis process. J. Therm. Anal. Calorim..

[77] Zhang Y., Liu Z., Hui L., Wang H. (2019). Diols as solvent media for liquefaction of corn stalk at ambient pressure. BioResources.

[78] Shawal N.N., Murtala A.M., Adilah A.K., Hamza U.D. (2012). Identification of Functional Groups of Sustainable Bio-Oil Substrate and its Potential for Specialty Chemicals Source. Adv. Mater. Res..

